# Factors Influencing the Choice of Glucose-Lowering Medications Among Physicians Treating Patients With Type 2 Diabetes

**DOI:** 10.7759/cureus.53844

**Published:** 2024-02-08

**Authors:** Mohammed E AlSofiani, Danah Z AlHalees, Joud A Aljebreen, Joud A Abu Dahesh, Ghada S Bamogaddam, Ghaida M AlBraithen, Anwar Jammah

**Affiliations:** 1 Endocrinology and Diabetes, King Saud Medical City, Riyadh, SAU; 2 Internal Medicine/Adult Diabetes and Endocrinology, King Saud University Medical City, Riyadh, SAU; 3 Endocrinology, Diabetes, and Metabolism, King Saud University, Riyadh, SAU

**Keywords:** treatment choices, type 2 diabetes, renal disease, cardiovascular disease, glucose-lowering agent

## Abstract

Background

The factors considered by physicians when prescribing a glucose-lowering agent to patients with type 2 diabetes (T2D) in real-world settings are not necessarily consistent with those recommended by clinical practice guidelines. Here, we identify the major factors that drive physicians’ selection of glucose-lowering agents in the real world and how these factors may differ by physician’s specialty.

Methods

A web-based survey was conducted among 135 physicians who manage patients with T2D in Saudi Arabia. Physicians were categorized according to their specialty into "specialists" (endocrinologists and/or diabetologists) and "generalists" (internists, family physicians, and primary care physicians). Physicians were asked about the type of glucose-lowering medication that they would typically prescribe in certain clinical scenarios and what factors drive such a selection.

Results

Sulfonylurea remains the most frequently prescribed second-line agent, as an add-on to metformin, according to 50% of the physicians surveyed. Most physicians (89%) reported prescribing glucagon-like peptide 1 receptor agonists (GLP-1RA) to less than half of their patients with T2D and ischemic heart disease; over two-thirds reported prescribing sodium-glucose cotransporter 2 inhibitors (SGLT-2i) to less than half of their patients with T2D and heart failure. When prescribing GLP-1RAs, the cost was a “major consideration” by 75% and 65% of the specialists and generalists, respectively. Likewise, when prescribing SGLT-2i, the cost was a major consideration by 57% and 71% of the specialists and generalists, respectively. Several other factors differed between the generalists and specialists when prescribing thiazolidinedione* *(TZD), sulfonylurea, dipeptidyl peptidase 4 (DPP-4) inhibitors, GLP-1RAs, and SLGT-2i, but not insulin.

Conclusion

Our findings highlight several challenges faced by physicians in the real world that may prevent them from adopting the latest evidence-based guidelines when managing patients with T2D. Health policies to increase accessibility to novel glucose-lowering agents, particularly for patients with T2D and cardiovascular/renal diseases, are needed.

## Introduction

According to the World Health Organization, Saudi Arabia ranks second in the Middle East and seventh globally for diabetes prevalence. Diabetes is linked to high mortality and vascular complications, becoming a major cause of medical complications and death in Saudi Arabia. However, research on diabetes in Saudi Arabia is notably lacking compared to developed countries [1].

There are over 10 classes of glucose-lowering agents currently available to treat patients with type 2 diabetes (T2D). Over the past two decades, newer classes of glucose-lowering agents have emerged, including glucagon-like peptide 1 receptor agonists (GLP-1RA), sodium-glucose cotransporter 2 inhibitors (SGLT-2i), and dual GLP-1/GIP agonists. In addition to improving glycemic control, these novel agents promote weight reduction and provide cardiovascular and/or renal protection along with other health-related benefits [2, 3]. Significant updates from the ADA and EASD joint recommendations in 2018 include the endorsement of SGLT-2i for T2DM patients with heart failure, particularly those with reduced ejection fraction, and the consideration of GLP-1RAs for high-risk T2DM patients without established CVD. Additionally, GLP-1RAs show notable benefits in reducing major adverse cardiovascular events for T2DM patients with established atherosclerotic CVD [4]. Moreover, they have a unique profile of adverse events which makes them not suitable for all people with T2D (PWT2D). The wide range of glucose-lowering medications and factors that physicians need to consider as they prescribe these medications have led to considerable variations in physicians’ clinical practice when managing PWT2D in real-world settings [5]. 

Several practice guidelines have been published by professional societies to guide clinicians through the process of selecting glucose-lowering agents when managing PWT2D. Many of these guidelines are updated regularly to incorporate the latest scientific evidence. Novel glucose-lowering agents are often prioritized in the proposed treatment algorithms in these guidelines. However, these algorithms are not always adaptable in the real world. Day-to-day clinical practices, patients’ characteristics, lifestyles, preferences, and goals of therapy as well as medications’ availability and cost vary remarkably across countries and clinics within the same country. Such factors are often not addressed in clinical practice guidelines, although they play a major role in medication selection in the real world. As a result, healthcare systems and policymakers are often faced with challenges as they try to adopt these evidence-based guidelines into the daily practice of physicians. 

Identifying factors that clinicians consider when selecting a glucose-lowering agent in their daily practice will have substantial implications for physicians, policymakers, healthcare systems, and PWT2D. In this study, we aim to identify patient-related, physician-related, and medication-related factors that physicians consider as a “major factor” when prescribing a glucose-lowering agent. In addition, we aim to identify how these factors may differ between "specialists" (endocrinologists and/or diabetologists) and "generalists" (internists, family physicians, and primary care physicians). 

## Materials and methods

Study participants

This is a cross-sectional, web-based, survey of physicians who manage PWT2D in Saudi Arabia. Physicians were selected through a convenient sampling technique. An online survey was sent to endocrinologists and diabetologists (i.e. "specialists"), and general internists, family physicians, and primary care physicians (i.e. "generalists") between February 5th and 20th, 2021. Physicians who treat pediatric patients only were excluded as they may have limited experience with some of the glucose-lowering agents. Out of 182 physicians approached, 135 (74%) physicians completed the survey. 

Study variables

Before dissemination to the study participants, the survey was piloted on eight physicians and updated according to their feedback. The survey consisted of four sections: (a) online informed consent, (b) physician’s demographics, specialty, and setting of clinical practice (public vs private vs both), (c) physician’s prior experience with each glucose-lowering agent, and (d) factors that the physician considers when prescribing each glucose-lowering agent along with the level of the impact of those factors on the physician’s choice of medication. For each medication, physicians were asked to rate a list of patient-related, physician-related, and medication-related factors that they would consider in their decision to select that particular medication. Physicians were asked to rate those factors as “major consideration”, “minor consideration”, or “not a consideration”. The factors listed in the survey included patient-related factors (patient’s age, gender, BMI, hemoglobin A1C, fasting glucose, liver function test, renal function status, patient’s desire to avoid insulin, potential cardiovascular and/or renal benefits, and others); physician-related factors (physician’s age, gender, specialty, and number of patients seen per week), and medication-related factors (effect of the medication on insulin resistance, effect of the medication on beta-cell function, risk of hypoglycemia, cardiovascular benefits, effect of the medication on renal function, effect of the medication on lipid panel, tolerability, and cost).

The study was approved by the Institutional Review Board at the College of Medicine, King Saud University, and informed consent was obtained from the study participants. 

Statistical analysis

We conducted our analysis using SPSS v. 26 (IBM Corp., Armonk, NY). Descriptive statistics (frequencies and percentages) were used. The chi-square test was applied for the difference estimate. A p-value of less than 0.05 was used as a statistical significance value of the results.

## Results

Characteristics of study participants

**Figure 1:**
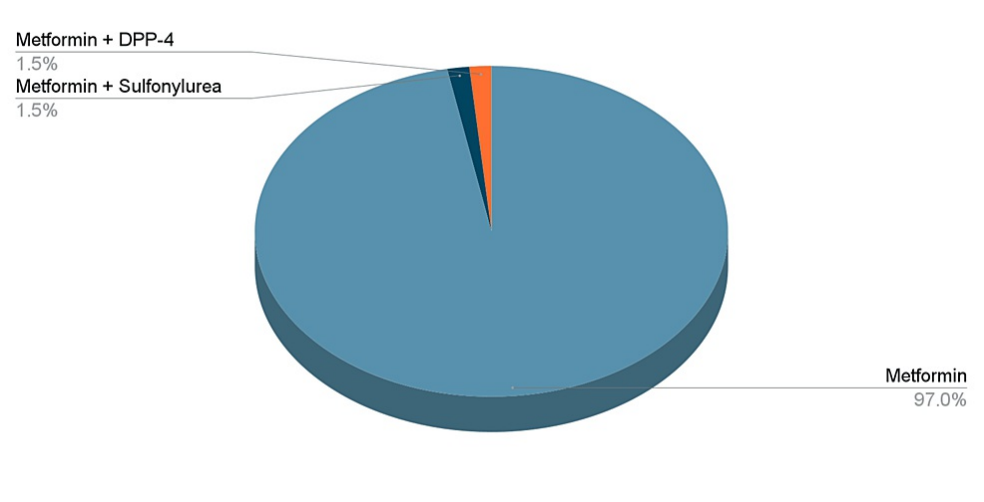
Most commonly prescribed glucose-lowering agent as a first-line agent DPP-4: dipeptidyl peptidase 4

**Figure 2:**
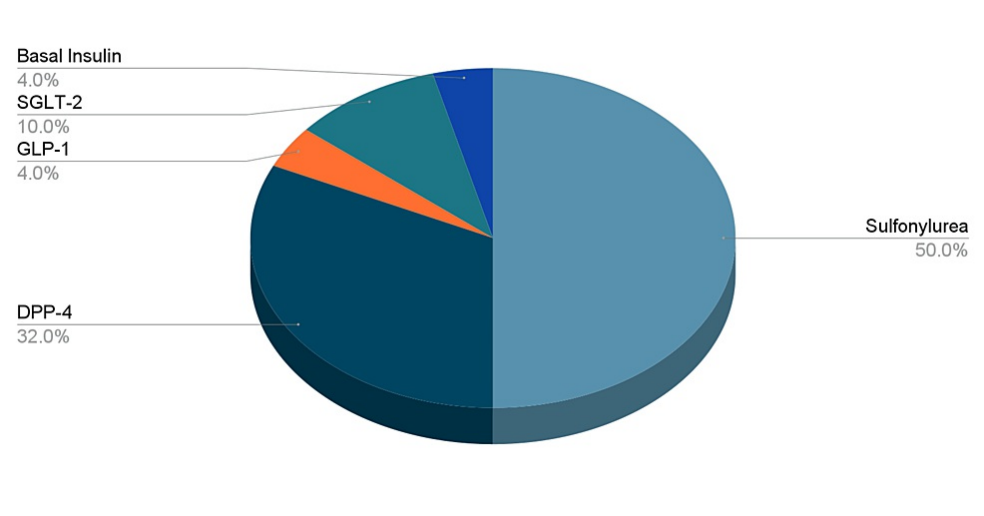
Most commonly prescribed glucose-lowering agent as a second-line agent SGLT-2: sodium glucose cotransporter 2; GLP-1: glucagon-like peptide 1; DPP-4: dipeptidyl peptidase 4

Out of the 135 physicians who completed the survey, 92 (68%) were women, 28 (20.7%) were older than 50 years old, 91 (67%) were generalists, and 44 (33%) were specialists (Table 1). Metformin was the first-line treatment agent for 131 (97%) of the clinicians, followed by metformin + dipeptidyl peptidase 4 (DPP-4) inhibitors for two (1.5%), and metformin + sulfonylurea for two(1.5%) (Figure 1). The most prescribed second-line agent (as an add-on to metformin) was sulfonylurea as reported by 68 (50%) of clinicians, followed by DPP-4 inhibitors 46 (33%). SGLT-2i and GLP-1RA were prescribed as second-line agents by only 15 (10%) and six (4%) clinicians, respectively (Figure 2).

**Table 1:** Distribution of demographic characteristics of study subjects (n=135) and their most commonly prescribed first- and second-line agents

Demographic characteristics	All (n=135); N(%)	Specialists (n=44); N(%)	Generalists (n=91); N(%)
Age			
< 30 Years	25 (18.5)	1 (2.27)	24 (26.37)
30-40 Years	48 (35.6)	10 (22.73)	38 (41.76)
41-50 Years	34 (25.2)	21 (47.73)	13 (14.29)
>50 Years	28 (20.7)	12 (27.27)	16 (17.58)
Gender			
Women	92 (68)	22 (50)	70 (76.92)
Men	43 (32)	22 (50)	21 (23.08)
Number of patients seen (patient/week)			
<10	29 (21.48)	3 (6.82)	26 (28.57)
10-30	45 (33.33)	13 (29.55)	32 (35.16)
31-60	20 (14.81)	6 (13.64)	14 (15.38)
>60	41 (30.37)	22 (50)	19 (20.88)

Utilization of novel glucose-lowering agents in PWT2D and ischemic heart disease and/or chronic kidney disease (CKD)

**Figure 3:**
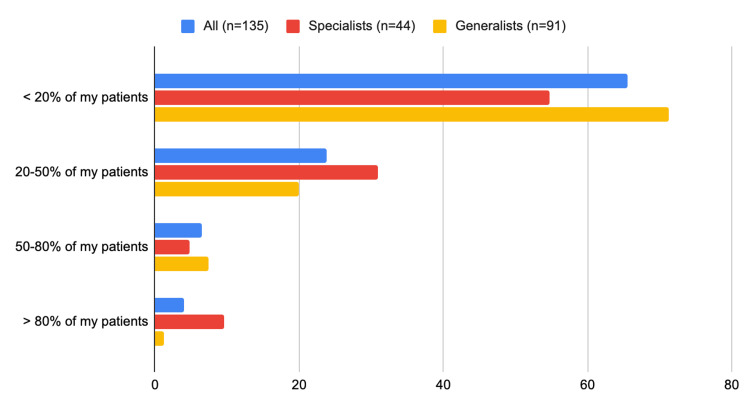
What is the percentage of PWT2D who also have ischemic heart disease for whom you prescribe GLP-1RAs? PWT2D: people with type 2 diabetes; GLP-1RA: glucagon-like peptide 1 receptor agonist

**Figure 4:**
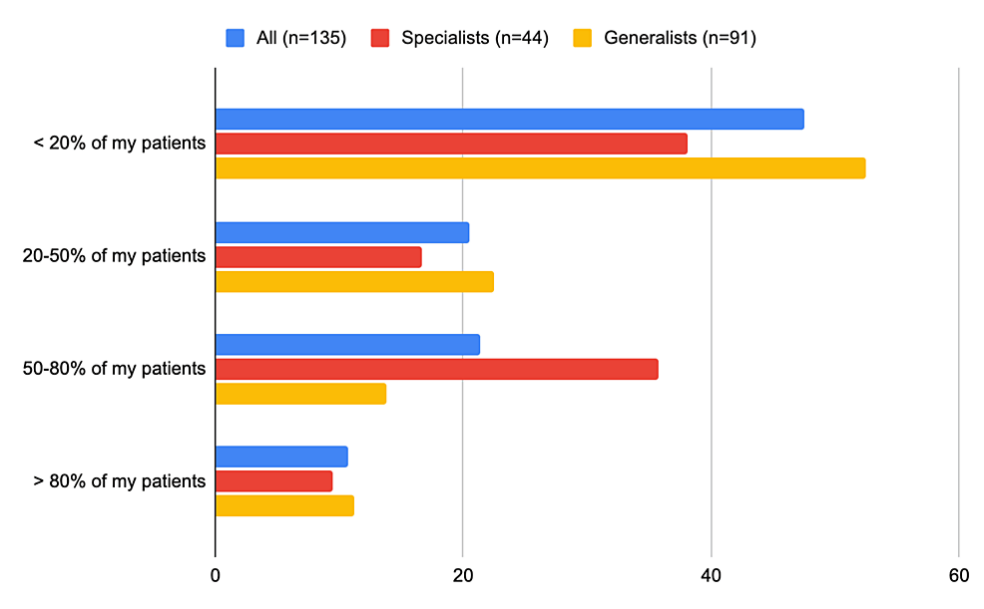
What is the percentage of your PWT2D who also have ischemic heart disease, heart failure, or chronic kidney disease for whom you prescribe SGLT-2i PWT2D: people with type 2 diabetes; SGLT-2i: sodium-glucose cotransporter 2 inhibitor

In total, 90 (two-thirds) of all physicians reported prescribing GLP-1RAs to only <20% of their patients with T2D and ischemic heart disease (65 (71%) of the generalists versus 24 (55%) of the specialists, p=0.06) (Figure 3, Table 2). Likewise, 65 (48%) of all physicians reported prescribing SGLT-2i to only <20% of their patients with T2D who also have ischemic heart disease, heart failure, or chronic kidney disease (48 (53%) of the generalists versus 17 (38%) of the specialists (p=0.05)) (Figure 4).

**Table 2:** Proportions of physicians who were aware of the cardiovascular benefits and renal benefits of GLP-1RAs and SGLT-2 inhibitors and rated them as a "major consideration" when selecting a medication The p-value was calculated using the chi-square test; a p-value less than 0.05 was considered statistically significant. GLP-1RA: glucagon-like peptide 1 receptor agonist; SGLT-2i: sodium glucose cotransporter 2 inhibitor

Parameters	Specialists (n=44) N(%)	Generalists (n=91) N(%)	P-value*
GLP-1RA			
Cardiovascular benefit	40 (90.91)	62 (68.13)	<0.01
Renal benefit	31 (70.45)	45 (49.4)	0.02
SGLT-2i			
Cardiovascular benefit	42 (95.45)	63 (69.23)	<0.01
Renal benefit	42 (95.45)	54 (59.43)	<0.01
Women	92 (68)	22 (50)	70 (76.92)
Men	43 (32)	22 (50)	21 (23.08)
Number of patients seen (patient/week)			
<10	29 (21.48)	3 (6.82)	26 (28.57)
10-30	45 (33.33)	13 (29.55)	32 (35.16)
31-60	20 (14.81)	6 (13.64)	14 (15.38)
>60	41 (30.37)	22 (50)	19 (20.88)

Top 3 factors considered by physicians when they prescribe each glucose-lowering agent

Thiazolidinedione (TZD)

**Figure 5:**
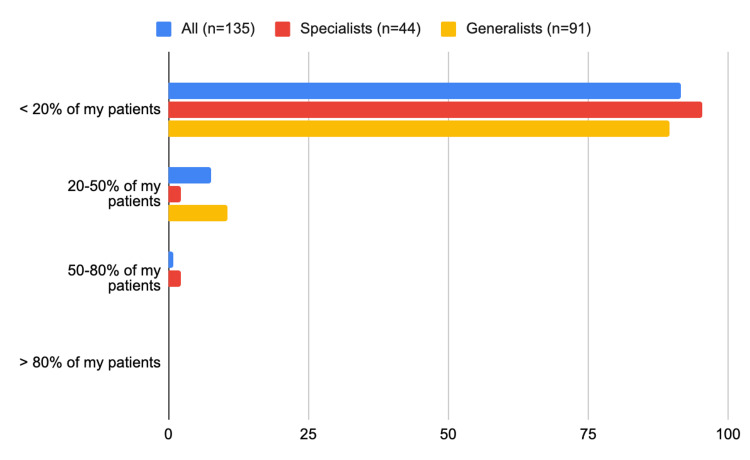
Frequency of prescribing each glucose-lowering agent by specialists versus generalists: What is the percentage of PWT2D for whom you prescribe TZD? PWT2D: people with type 2 diabetes; TZD: thiazolidinedione

The notable risk of heart failure (HF) associated with TZDs [6] has led to a significant observation: both generalists and specialists did not prioritize cardiovascular benefits when selecting TZD as a treatment option. The top three factors that influenced all physicians' decision to prescribe TZD were the patient’s liver function (n=86; 64%), TZD’s effect on insulin resistance (n=72; 53%), and the patient’s fasting glucose level (n=49; 36%). TZD’s effect on insulin resistance was a major consideration for 33 (75%) of the specialists compared to only 38 (42%) of the generalists (p<0.01). Whereas, fasting glucose level was a major consideration for only 10 (21%) of the specialists compared to 40 (44%) of the generalists (p=0.03) (Figure 5, Table 3).

**Table 3:** Comparison of factors’ major considerations when prescribing diabetes mellitus medications between specialists and generalists *P-value calculated using chi-square test; p-value less than 0.05 is considered statistically significant. TZD: thiazolidinedione; SU: sulfonylurea; DPP-4: dipeptidyl peptidase 4; GLP-1RA: glucagon-like peptide 1 receptor agonist; SGLT-2i: sodium glucose cotransporter 2 inhibitor; BMI: body mass index

Major considerations	All (%) N(%)	Specialists (%) N(%)	Generalists (%) N(%)	P-value*
TZD				
Effect on Insulin resistance	71 (52.5)	33 (75)	38 (41.8)	0.001
Patient's fasting glucose	49 (36.3)	9 (20.5)	40 (43.9)	0.028
Patient's liver function	86 (63.7)	34 (77.3)	52 (57.1)	0.061
SU				
Patient's fasting glucose	87 (64.4)	19 (43.2)	68 (74.7)	<0.001
Patient's side effect complaints	61 (45.9)	12 (28.6)	49 (53.9)	0.004
Effect on beta-cell function	66 (48.9)	27 (61.4)	39 (42.9)	0.102
DPP-4 inhibitors				
Patient’s fasting glucose	60 (44.4)	13 (29.6)	47 (51.7)	0.011
Patient’s liver function	70 (51.9)	27 (61.4)	43 (47.3)	0.12
Renal effect	70 (51.9)	27 (61.4)	43 (47.3)	0.12
GLP-1RA				
Cardiovascular benefit	102 (75.6)	40 (90.9)	62 (68.1)	0.011
Renal effect	76 (56.3)	31 (70.5)	45 (49.5)	0.009
Risk of hypoglycemia	49 (36.3)	21 (47.7)	28 (30.8)	0.06
GLP-1's cost	92 (68.15)	33 (75)	59 (64.84)	0.24
SGLT-2i				
Renal effect	96 (71.1)	42 (95.5)	54 (59.3)	<0.001
Cardiovascular benefit	105 (77.8)	42 (95.5)	63 (69.2)	0.002
Patient's BMI	94 (69.6)	36 (81.8)	58 (63.7)	0.049
Risk of hypoglycemia	79 (58.5)	31 (70.5)	48 (52.8)	0.05
SGLT-2's cost	83 (61.48)	31 (70.45)	52 (57.14)	0.14
Insulin				
Patient's side effect complaints	89 (65.9)	34 (77.3)	55 (60.4)	0.05
Patient’s BMI	93 (68.9)	34 (77.3)	59 (64.8)	0.14
Patient’s liver function	69 (51.1)	19 (43.2)	50 (55)	0.2

Sulfonylurea

**Figure 6:**
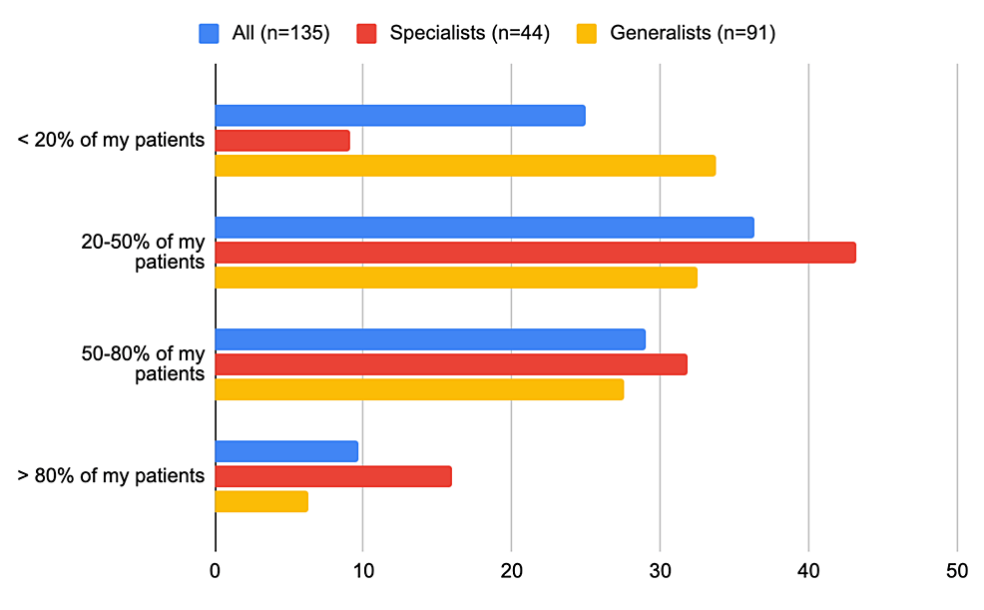
Frequency of prescribing each glucose-lowering agent by specialists versus generalists: What is the percentage of PWT2D for whom you prescribe SU? PWT2D: people with type 2 diabetes; SU: sulfonylurea (SU)

The top three factors that influenced all physicians' decision to prescribe sulfonylurea were the patient’s fasting glucose level (n=87; 64%), the patient’s concern about side effects (n=62; 46%), and the effect of sulfonylurea on beta-cell function (n=66; 49%). The patient’s fasting glucose level was a major consideration for 19 (43%) of the specialists compared to 68 (75%) of the generalists (p<0.01), whereas, patient concern about side effects was a major consideration for 13 (29%) of the specialists compared to 49 (54%) of the generalists (p<0.01) (Figure 6, Table 3).

DPP-4i

**Figure 7:**
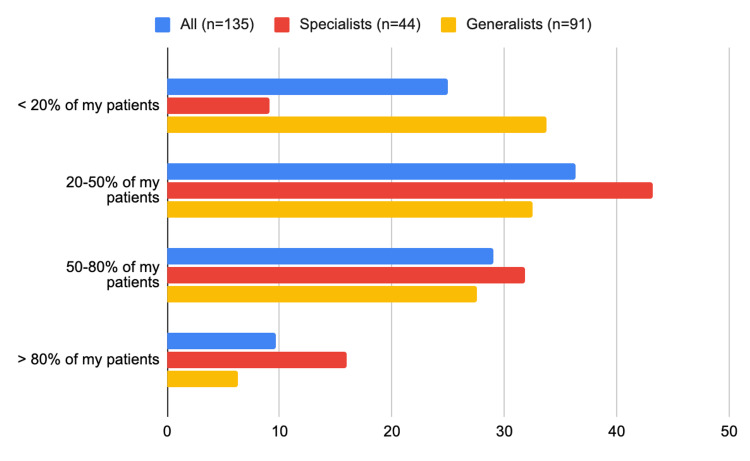
Frequency of prescribing each glucose-lowering agent by specialists versus generalists: what is the percentage of PWT2D for whom you prescribe DPP-4 Inhibitors? DPP-4: dipeptidyl peptidase 4

The top three factors that influenced all physicians' decision to prescribe DPP-4i were the patient's fasting glucose level (n=59; 44%), the patient’s liver function (n=70; 52%), and the patient’s renal function (n=70; 52%). The patient’s fasting glucose level was a major consideration for 13 (30%) of the specialists compared to 47 (52%) of the generalists (p=0.01). Whereas, the patient's liver function was a major consideration for 27 (61%) of specialists compared to 43 (47%) of generalists (p=0.12) (Figure 7, Table 3).

GLP-1RA

The top three factors that influenced all physicians' decision to prescribe GLPA-1RAs were the potential cardiovascular benefit (n=103; 76%), the potential renal benefit (n=76; 56%), and the risk of hypoglycemia (n=49; 36%). The cardiovascular benefit of GLP-1RA was a major consideration for 40 (91%) of the specialists compared to 62 (68%) of the generalists (p<0.01), whereas, the renal benefit of GLP-1RA was a major consideration for 31 (70%) of the specialists compared to 45 (49%) of the generalists (p<0.01) (Table 3).

SGLT-2i

The top three factors that influenced all physicians' decision to prescribe SGLT-2i were its cardiovascular benefits (n=106; 78%), renal benefit (n=96; 71%), and patient’s BMI (n=95; 70%). Cardiovascular benefit was a major consideration for 42 (95%) of the specialists compared to 63 (69%) of the generalists (p<0.01). Whereas, the renal benefit of SGLT-2i was a major consideration for 42 (95%) of the specialists compared to 54 (59%) of the generalists (p<0.01). Patient’s BMI was a major consideration for 36 (82%) of the specialists compared to 58 (64%) of the generalists (p=0.049) (Table 3).

Insulin

The top three factors that influenced all physicians' decision to prescribe insulin were the patient’s BMI (as reported by 93 (69%) of the physicians), the patient’s liver function (n=69; 51%), and the patient's side effects complaints 89 (66%). Patient side effect complaint was a major consideration for 34 (77%) of the specialists compared to 55 (60%) of the generalists (p=0.05), whereas the patient’s BMI was a major consideration for 34 (77%) of specialists compared to 59 (65%) of generalists (p=0.14) (Table 3).

Cost

**Figure 8:**
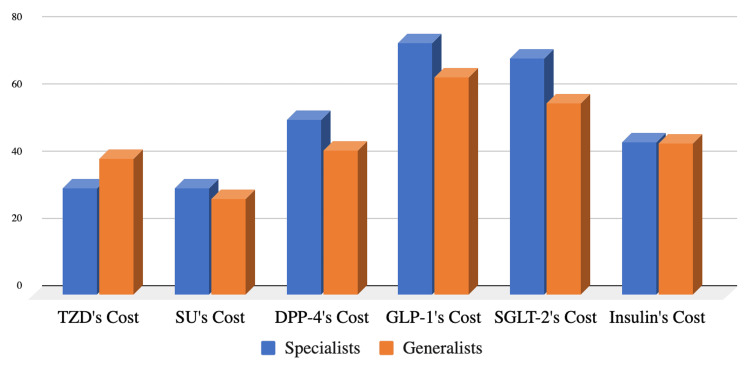
Percentages of physicians who think cost is a major consideration when prescribing the following glucose-lowering agents TZD: thiazolidinedione; SU: sulfonylureas; DPP-4: dipeptidyl peptidase 4; GLP-1RA: glucagon-like peptide 1 receptor agonist; SGLT-2: sodium-glucose cotransporter 2

Physicians prioritize cost considerations when prescribing oral hypoglycemic medications. SGLT-2i and GLP-1RAs, while highly effective, present a higher financial burden, making them the most considered in terms of cost. DPP4 inhibitors closely follow with an intermediate pricing range. Conversely, sulfonylurea stands out as the least considered medication for cost, emerging as a preferred and cost-effective choice for individuals managing diabetes (Figure 8).

## Discussion

Our study is the first to evaluate factors considered by physicians in Saudi Arabia when they prescribe a glucose-lowering agent for PWT2D. The healthcare system in Saudi Arabia is now amid a major transformation from being a completely public/governmental-based healthcare system (where services are paid for by the government) to a private healthcare system (where services are paid for by non-governmental payers such as healthcare insurance) [7,8]. This transformation in the healthcare system has created unique dynamics in the healthcare services provided to PWT2D and provided us with a unique opportunity to examine the impact of such transformation on the prescribing practices of physicians who manage those patients. For instance, the accessibility to various glucose-lowering agents and their availability and affordability are changing because of this healthcare transformation. Not surprisingly, this has impacted the factors considered by physicians when selecting a glucose-lowering medication in Saudi Arabia as highlighted in our study.

Metformin remains the most commonly prescribed first-line glucose-lowering agent by specialists and generalists. It is commonly recommended as the first-line treatment for diabetes due to its low hypoglycemia risk, strong antihyperglycemic efficacy, weight-loss or weight-neutral benefits, and cost-effectiveness. Its cardiovascular safety profile is well-supported compared to sulfonylureas. However, caution is advised for individuals with an increased risk of lactic acidosis, such as those with chronic kidney or hepatic disease [9]. This result is consistent with most clinical practice guidelines and does not seem to have been impacted by the current healthcare transformation taking place in Saudi. A recent study in Japan identified age, BMI, disease duration, and glycemic control as key factors in determining the type of glucose-lowering agent utilized by clinicians as the initial monotherapy in PWT2D [10]. Another study, in the United States, reported qualitative factors, such as patient adherence, are being prioritized by clinicians over quantitative factors such as the patient’s hemoglobin A1C when prescribing a glucose-lowering agent [3]. In another study in Japan, DPP-4 inhibitors were the first-line treatment for T2D preferred by most clinicians, followed by metformin [11]. Our findings are consistent with another previous study in Saudi Arabia, in 2020, that also identified metformin, with or without sulfonylurea, as the most prescribed first-line treatment for PWT2DM, regardless of the healthcare setting [12]. In our study, however, we further explored the physicians’ perspective on why that is the case and how they typically select certain glucose-lowering agents for PWT2D. 

Interestingly, sulfonylurea remains the second most commonly prescribed glucose-lowering agent in Saudi despite the evolution of novel glucose-lowering agents (such as GLP-1RA and SGLT-2i) which provide comparable glucose-lowering efficacy along with additional cardiovascular and/or renal benefits. From 2018 to 2020, less than one in eight individuals with T2D, who met the criteria outlined in evidence-based guidelines and professional society recommendations, utilized GLP-1RA and SGLT-2i. The one-year fill rates for these medications hovered around 50%. This suboptimal and inconsistent utilization undermines the potential long-term health benefits of these drugs, especially during a time when indications for their use are expanding [13]. Over two-thirds of the physicians in our study rated the cardiovascular and renal benefits of GLP-1RA and SGLT-2i as a “major consideration” when prescribing these agents. Yet, they reported utilizing GLP-1RA in less than 20% of their PWT2D and ischemic heart disease and reported prescribing SGLT-2i in less than 20% of their PWT2D and chronic kidney disease. The underutilization of these novel glucose-lowering agents by the physicians in our study is likely attributed to the higher cost of, and limited accessibility to, these medications. This is supported by what most physicians in our study reported as cost being a “major consideration” when they prescribe GLP-1 agonists or SGLT-2I. Moreover, a lack of awareness about the clinical benefits of these novel agents, especially among generalists, seems to be another barrier to the utilization of these agents. 

The cost of GLP-1RA and SGLT-2i remains a major barrier to the wide utilization of these agents in patients who need them the most including those with T2D and cardiovascular and/or renal disease. This is highlighted in our study by the discrepancy between the high proportion of physicians who are aware of the cardiorenal benefits of GLP-1RA and SGLT-2i and the low rate of utilization of these agents in PWT2D and cardiovascular and/or renal diseases as reported by the same physicians. The cost of these medications was reported as a major consideration by most of these physicians.

Our study has several strengths. This is the first study, to our knowledge, to explore the discrepancy between the recommendations by widely accepted clinical guidelines and the actual physicians’ practice in a real-world setting. Moreover, we surveyed both specialists and generalists who are frequently managing PWT2D in their clinics to formulate a better understanding of the current prescribing practices in the real-world setting. Our findings have several clinical and health policy implications. Addressing barriers to the utilization of novel glucose-lowering agents in PWT2D who need them the most, as highlighted in our study, should be a public health priority. In addition, raising awareness about the cardiorenal benefits of GLP-1RA and SGLT-2i, particularly among generalists, as well as studying the cost-effectiveness of novel glucose-lowering agents are needed to address the current clinical care gaps. 

We surveyed physicians from various institutions within Saudi Arabia; and therefore, our findings cannot be generalized to healthcare systems in other countries. Moreover, the relatively small sample size of our study may have limited our ability to detect smaller differences between the specialists and generalists in some of the factors that they consider when prescribing glucose-lowering agents. Finally, GLP-1/GIP dual agonists were not available in Saudi during the study period; therefore, we did not include any questions about this class of medication in our survey. 

## Conclusions

Our study highlights the current gaps in the knowledge of non-specialists about novel glucose-lowering agents and their cardiovascular and renal benefits, along with other challenges that specialists and non-specialists face as they consider those novel agents for the management of PWT2D in the real-world setting. These challenges may explain the limited adoption of some of the clinical guideline recommendations in the real-world setting in many parts of the world. Healthcare policies to increase physicians’ awareness about, and patients’ accessibility to, novel glucose-lowering medications, are needed. Moreover, there is a need for local clinical practice guidelines that take into consideration the dynamics of the local healthcare system and barriers to selection of certain glucose-lowering medications. The development of such localized guidelines should be prioritized over the adoption of international guidelines that may not be applicable in some parts of the world.

## References

[1] Al Dawish MA, Robert AA, Braham R, Al Hayek AA, Al Saeed A, Ahmed RA, Al Sabaan FS (2016). Diabetes mellitus in Saudi Arabia: a review of the recent literature. Curr Diabetes Rev.

[2] Del Olmo-Garcia MI, Merino-Torres JF (2018). GLP-1 receptor agonists and cardiovascular disease in patients with type 2 diabetes. J Diabetes Res.

[3] Lopaschuk GD, Verma S (2020). Mechanisms of cardiovascular benefits of sodium glucose co-transporter 2 (SGLT2) inhibitors: a state-of-the-art review. JACC Basic Transl Sci.

[4] Ma CX, Ma XN, Guan CH, Li YD, Mauricio D, Fu SB (2022). Cardiovascular disease in type 2 diabetes mellitus: progress toward personalized management. Cardiovasc Diabetol.

[5] Grant RW, Wexler DJ, Watson AJ, Lester WT, Cagliero E, Campbell EG, Nathan DM (2007). How doctors choose medications to treat type 2 diabetes: a national survey of specialists and academic generalists. Diabetes Care.

[6] Asleh R, Sheikh-Ahmad M, Briasoulis A, Kushwaha SS (2018). The influence of anti-hyperglycemic drug therapy on cardiovascular and heart failure outcomes in patients with type 2 diabetes mellitus. Heart Fail Rev.

[7] Alasiri AA, Mohammed V (2022). Healthcare transformation in Saudi Arabia: an overview since the launch of Vision 2030. Health Serv Insights.

[8] Rahman R (2020). The privatization of health care system in Saudi Arabia. Health Serv Insights.

[9] Schroeder EB (2022). Management of type 2 diabetes: selecting amongst available pharmacological agents. Endotext.

[10] Fujihara K, Igarashi R, Matsunaga S (2017). Comparison of baseline characteristics and clinical course in Japanese patients with type 2 diabetes among whom different types of oral hypoglycemic agents were chosen by diabetes specialists as initial monotherapy (JDDM 42). Medicine (Baltimore).

[11] Murayama H, Imai K, Odawara M (2018). Factors influencing the prescribing preferences of physicians for drug-naive patients with type 2 diabetes mellitus in the real-world setting in Japan: insight from a web survey. Diabetes Ther.

[12] Al-Rubeaan K, Bana FA, Alruwaily FG (2020). Physicians' choices in the first- and second-line management of type 2 diabetes in the Kingdom of Saudi Arabia. Saudi Pharm J.

[13] Nargesi AA, Clark C, Chen L (2022). Patterns of medication use and prescription fills for cardioprotective anti-hyperglycemic agents in the United States [PREPRINT]. medRxiv.

